# A morphological and molecular study of *Anaplasma phagocytophilum *transmission events at the time of *Ixodes ricinus *tick bite

**DOI:** 10.1186/1751-0147-52-43

**Published:** 2010-06-17

**Authors:** Erik G Granquist, Mona Aleksandersen, Karin Bergström, Stephen J Dumler, Wenche O Torsteinbø, Snorre Stuen

**Affiliations:** 1 Department of Production Animal Clinical Sciences, Section of Small Ruminant Research, Norwegian School of Veterinary Science, Sandnes, N-4325 Norway; 2 Department of Basic Sciences and Aquatic Medicine, Norwegian School of Veterinary Science, Oslo, N-0033 Norway; 3 National Veterinary Institute, Uppsala 75189, Sweden; 4 Department of Pathology, Division of Medical Microbiology, The Johns Hopkins Hospital, Baltimore, MD 21205 USA

## Abstract

**Background:**

*Anaplasma phagocytophilum *is the causative agent of human granulocytic anaplasmosis (HGA) in humans and tick-borne fever (TBF) in ruminants. The bacterium invades and replicates in phagocytes, especially in polymorphonuclear granulocytes.

**Methods:**

In the present study, skin biopsies and ticks (*Ixodes ricinus*) were collected from tick feeding lesions on 38 grazing lambs between two and three weeks after access to pastures. The histopathological changes associated with tick bites and *A. phagocytophilum *infection, were described. In addition the skin biopsies were examined by immunohistochemistry. Furthermore, samples from blood, skin biopsies and ticks were examined by serology, PCR amplification of *msp2 *(*p44*), genotyping of *rrs *(16S rRNA) variants, and compared with the results obtained from histological and immunohistochemical investigations.

**Results:**

Tick bites were associated with chronic and hyperplastic inflammatory skin lesions in this study. *A. phagocytophilum *present in skin lesions were mainly associated with neutrophils and macrophages. Bacteria were occasionally observed in the Tunica media and Tunica adventitia of small vessels, but were rarely found in association with endothelial cells. PCR and genotyping of organisms present in blood, ticks and skin biopsies suggested a haematogenous and a local spread of organisms at the tick attachment sites.

**Conclusions:**

The present study describes different aspects of *A. phagocytophilum *infection at the site of tick bite, and indicates that *A. phagocytophilum *rarely associates with endothelium during the early pathogenesis of infection.

## Introduction

*Anaplasma phagocytophilum *is recognized as the causative agent of Human Granulocytic Anaplasmosis (HGA) in humans and tick-borne fever (TBF) in ruminants [1-3]. Although self-limiting in sheep, immune suppression with infection often results in secondary infections that complicate the clinical picture [4]. TBF is of growing concern from the production and animal welfare perspectives in the sheep industry [5].

*A. phagocytophilum *is known to primarily infect and propagate in polymorphonuclear leucocytes (PMN) [6-8]. Its strict intracellular location provides a mechanism for evading host defences, and also promotes chemotactic mechanisms (IL-8) that assist the attraction of neutrophils to the tick bite site [9]. Degranulation of neutrophils at the tick bite site increases the permeability of blood vessels and increases the cellular infiltration of the area [10,11]. Because of the short-lived nature of circulating neutrophils, the role of these cells in establishing and maintaining infection has been questioned [10]. Earlier studies have suggested that cells other than PMN are involved in the early pathogenesis, since ticks do not directly tap the blood vessels and thus cannot directly deliver pathogens to circulating leukocytes [12-15].

Once inside the host cell however, a closed microenvironment structurally designed to protect vital processes within the cell, gives shelter from extracellular humoral and cellular immune responses [16-20]. Earlier studies in cell culture have shown that endothelial cells are capable of being infected with *A. phagocytophilum *and support infection *in vitro *[10,15,21].

The rationale of the present study was to examine the local skin inflammation, created during *A. phagocytophilum *infection, and if endothelial cells may act as *in vivo *host cells for *A. phagocytophilum *during natural infection in lambs. Skin biopsies were collected from tick attachment sites and examined by histology, immunohistochemistry, PCR amplification of *msp2 *(*p44*) and genotyping of *A. phagocytophilum *by PCR amplification and sequencing of *rrs *(16S rRNA gene). Blood samples were also examined for the presence of bacteraemia by PCR amplification and *rrs *(16S rRNA gene) genotyping of *A. phagocytophilum *in addition to indirect fluorescence antibody test (IFAT).

## Materials and methods

### Animals and sampling

Skin biopsies, EDTA blood and serum samples from 38 lambs of the Norwegian White breed from two flocks were collected in May and June of the 2006 and 2007 grazing seasons, in the Rogaland and Vest-Agder county of Norway, respectively. The lambs were 4-6 weeks old and the samples were collected between two and three weeks after the lambs were put to pastures that were previously known to be heavily infested with the sheep tick (*Ixodes ricinus*). The individual animals were selected for sampling based on the presence of at least two fresh tick bites. In addition, the rectal temperature was measured as an indicator of acute tick-borne fever [22]. If ticks were still attached, they were collected and stored unfixed on individual plastic tubes for later PCR amplification of *msp2 *(*p44*) to determine if they were infected by *A. phagocytophilum*. The wool in the tick bite area was sheared, and the skin surface was disinfected by 70% ethanol, before a subcutaneous ring block of local anaesthesia was laid around the tick bite (0.5-1.0 ml 2% Carbocain™, AstraZeneca). A punch biopsy knife (8 mm in diameter) was used for collection of the skin biopsies [23]. Two biopsies from the tick bite sites and one control biopsy at least 20 cm from other ticks or tick bites were collected from each lamb.

The biopsy wounds were closed by agraffe sutures. The skin biopsies were cut in two halves with sterile scalpels. One half was stored on Zamboni's fixative before histological processing and the other was kept on ice until frozen at -80°C for later DNA isolation. The experiment was approved by the National Animal Research Authority in Norway.

### Real time PCR for identification of positive samples, targeting *msp2 *(*p44*)

DNA was isolated from EDTA blood and skin biopsies, using a DNeasy^® ^Blood and Tissue kit (Qiagen GmbH, Hilden, Germany) according to protocols provided by the DNeasy^® ^Blood and Tissue Handbook (2006). DNA from ticks was isolated using the DNeasy^® ^Tissue kit (QIAGEN) for isolation from insects, according to protocols provided by the DNeasy^® ^Tissue kit Handbook (2004), with modifications as follows; The volume of Proteinase K was doubled and the incubation time with Proteinase K was extended to be 24 hours. The isolated DNA was diluted according to spectrum readings and final template volume was 5 μl containing 2.5 ng/*μ*l total DNA. PCR positive samples were detected by Real Time PCR using the Lightcycler^® ^480 (LC480) (Roche Diagnostics Meylan, France) with Fast Start MASTER^PLUS ^SYBR-green I Taq polymerase mix and fluorescence detection. The specific primers (Ap*msp2*f: 5'-ATG GAA GGT AGT GTT GGT TAT GGT ATT-3'and Ap*msp2*r: 5'-TTG GTC TTG AAG CGC TCG TA-3') were designed to amplify a 77 bp segment at the conserved N-terminal coding region of *msp2 *(*p44*)in the *A. phagocytophilum *genome [24]. Crossing points (CP) were determined by using the 2^nd ^derivative maximum method of the LightCycler^® ^Software 1.5.0 (Roche Diagnostics). The Cq (treshold cycle) was set to be 40 since *rrs *(16S rRNA gene) sequences (see below) were obtained from two tissues having CP values of 39 and 40, respectively. Further validation of *msp2 *(*p44*) amplicons was determined by melting point (Tm) analysis (range 82°C-83°C).

### Semi nested conventional PCR and sequencing of the 16S rRNA gene

DNA from blood and tissues were extracted according to the protocols described in the above section. A semi-nested PCR was conducted for amplification of *rrs *(16S rRNA gene) on a PTC-200 instrument (MJ Research) as previously described [25]. Briefly, an initial PCR was performed using primers *16S*-F5 (5'-AGTTTGATCATGGTTCAGA-3') and ANA-R4B (5'-CGAACAACGCTTGC-3') for amplification of a 507 bp fragment of *rrs *(16S rRNA gene) in *A. phagocytophilum*, followed by a semi-nested reaction with primers *16S*-F5 and ANA-R5 (5'-TCCTCTCAGACCAGCTATA-3') that produced a 282 bp fragment. Positive amplification was verified by agarose gel electrophoresis and amplified PCR products were sequenced directly, using Big Dye terminator cycle sequencing chemistry and capillary electrophoresis (ABI 310; Applied Biosystems). *A. phagocytophilum *variants were detected from visual inspection of the chromatograms [25].

### Haematology

Differential blood cell counts were performed on EDTA blood samples using the Advia 120 Automated Hematology Analyzer (Bayer Corporation, Tarrytown, NY, USA) for evaluation of neutropenia (< 0.7 × 10^9 ^cells/L).

### Serology

An indirect immunofluorescence antibody assay (IFA) was used to determine the polyvalent antibody titres to *A. phagocytophilum*. Briefly, two-fold dilutions of sera were added to slides precoated with antigen obtained from horses (formerly *Ehrlichia equi*) (Protatek, St. Paul. Minn., USA). Bound antibodies were visualized by fluorescein-isothiocyanate (FITC)-conjugated rabbit-anti-sheep immunoglobulin (Cappel, Organon Teknika, West Chester, PA, USA). Sera were screened for antibodies at dilution 1:40. If positive, the sera were further diluted and retested. A titre of 1.6 (log_10 _reciprocal of 40) or more was regarded as positive [26].

### Histology and immunohistochemistry

Skin samples fixed in Zamboni's fixative were routinely processed and embedded in paraffin. Tissue sections of 3 μm thickness were sectioned parallel to the tick bite and stained with haematoxylin and eosin for histological examination.

For immunohistochemistry (IHC), 3 μm thick sections were collected on Menzel-Gläser SuperFrost Ultra Plus^® ^slides (Braunschweig, Germany) and dried over night at 37°C. The sections were deparaffinised in xylene and rehydrated in graded alcohol solutions. The sections were treated with 0.1M citrate buffer (pH 6.0) at 92°C for 20 minutes in water bath or microwave oven for antigen retrieval and then cooled at room temperature for 30 minutes. After washing in distilled water, slides were placed in phosphate buffered saline (PBS) for equilibration. Endogenous peroxidase activity was inhibited by application of a methanol solution containing 1% H_2_O_2 _for 10 minutes, followed by washing in PBS and incubation for 20 minutes at room temperature with normal blocking serum (VECTASTAIN^® ^Elite kit) (Vector Laboratories, Burlingham, CA, USA), diluted 1:50 in PBS containing 5% bovine serum albumin (BSA/PBS).

The sections were incubated with either a monoclonal anti *A. phagocytophilum *antibody or a polyclonal rabbit anti *A. phagocytophilum *antibody. The primary antibodies were diluted 1:400 in 1% BSA/PBS and incubation was over night at 4°C. After washing in PBS, the sections were incubated with the biotinylated universal antibody from the kit according to the protocol provided by the producer (VECTASTAIN). The sections were further incubated for 30 minutes with the VECTASTAIN^® ^Elite ABC reagent after washing. Sections were exposed for the 3-amino-9-ethyl carbazole substrate (AEC) for 15 minutes and counterstained with non-alcoholic haematoxylin. Slides were washed three times in sterile water and mounted with poly vinyl alcohol (PVA).

## Results

### Examination of the animals

Twenty-three of 38 lambs (60.5%) had rectal temperatures above 40°C and the highest recorded temperature was 41.5°C. Thirteen lambs (34.2%) had neutropenia at the time of sampling and nine lambs (23.7%) had fever and neutropenia. The number of engorged ticks on the animals varied from one to more than 30 at the time of sampling. Skin biopsies were mostly collected from the axillary and inguinal regions as they were the most frequent tick attachment sites, registered. Tick bite sites showed typical mild erythema and local swelling.

### PCR amplification of *A. phagocytophilum msp2/p44 *in blood, skin biopsies and ticks

Thirty-three lambs (86.8%) were positive for *A. phagocytophilum *by PCR analysis of peripheral blood. Thirty-seven (97.4%) had one or more skin biopsies that were positive for *A. phagocytophilum *by PCR analysis. Seventy of 76 biopsies from tick attachment sites (92.1%) and 31 of 38 control biopsies (81.5%) were positive by PCR for *A. phagocytophilum *infection. A total of 68 ticks were collected from the lambs. Fifty-eight (85.3%) were positive for *A. phagocytophilum *by PCR. Two PCR positive ticks (2.9%) had a negative attachment site.

### Sequencing of *rrs *(16S rRNA gene)

Six different *rrs *(16S rRNA gene) isolates of *A. phagocytophilum *were encountered during the study, that were similar to GenBank acc. no. U02521, M73220, AF336220, AY035312, AJ242784, and a novel variant GU459257. All variants except AY035312 were collected from the flock in Vest-Agder county. The variants M73220, AJ242784 and AY035312 were collected from the flock in Rogaland county. A total of 38 partial *rrs *variant sequences were obtained from the tick bite biopsies. Nineteen of 38 sequences (50.0%) obtained from tick bite sites corresponded to the sequences obtained from the respective ticks. The sequences obtained from control biopsies and the blood samples were identical in all lambs where both sequences were obtained (N = 10) (data not shown). No direct relations between variants, serum titre and inflammatory changes were observed.

### Histology

**Figure 1:**
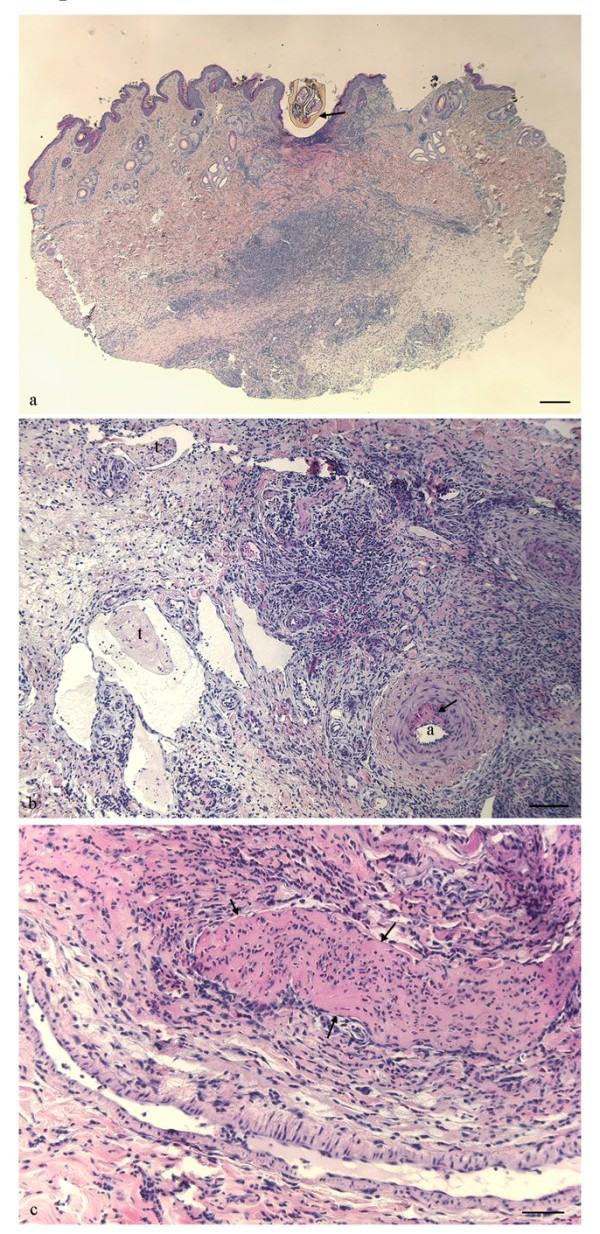
**Skin lesions in lambs naturally infected with *A. phagocytophilum***. a) Skin biopsy with an attached tick (arrow) and ulceration of epidermis. Inflammatory cell infiltrates are present in dermis and subcutis underneath the tick bite. [Haematoxylin and eosin. Bar = 300 μm.] b) Photomicrograph of dermis. Thrombi (t) are present in lymphatics and venules, and a focal necrosis is observed in the wall of an artery (a). Infiltration of leukocytes, moderate oedema and proliferation of fibrous tissue is found in dermis. [Haematoxylin and eosin. Bar = 100 μm.] c) Photomicrograph of dermis. A large thrombus is occluding the lumen of a vein (arrows). Infiltration of neutrophils, macrophages and lymphocytes are present in dermis. [Haematoxylin and eosin. Bar = 50 μm.]

Histological examination of biopsies from infected skin areas showed inflammatory lesions in 35 of the 38 lambs (92.1%). The majority of lambs (60.5%) had focal histopathologic changes, characterized by thickened epidermis, dermal fibroplasia and perivascular to diffuse infiltration of mixed leucocytes (Fig. 1a). Twelve lambs (31.6%) had milder changes with perivascular inflammatory cell infiltration in affected areas, whereas histopathological changes were not observed in three of the lambs. Focal ulcerations of the epidermis were observed in skin biopsies from 10 (26.3%) animals (Fig. 1a). The inflammatory exudate was composed of numerous neutrophils and eosinophils in addition to mast cells, lymphocytes and macrophages (Fig. 1b). The perivascular aggregates were mainly composed of mononuclear inflammatory cells. A substantial number of lambs, 14 out of 38, showed cellulitis with subcutaneous infiltration of neutrophils. Other lesions such as focal degeneration of dermal collagen (18.4%), vasculitis (10.5%), thrombosis of venules and lymphatics (15.8%) were observed (Fig 1c).

The different *rrs *(16S rRNA gene) variants of *A. phagocytophilum *seemed to produce similar pathological lesions. The control biopsies did not show inflammatory changes.

### Immunohistochemical examination, PCR and serology

**Figure 2:**
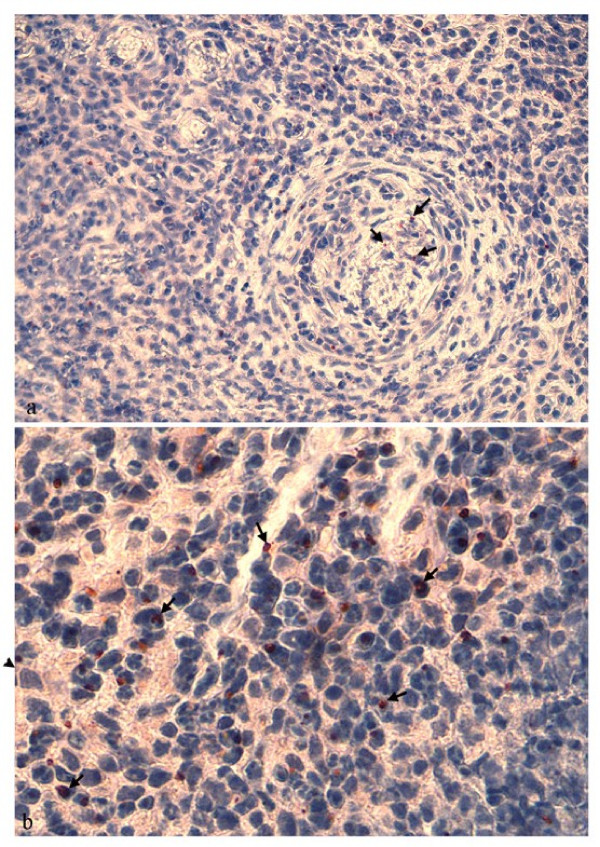
**Immunohistochemistry for *Anaplasma phagocytophilum *on skin tissue in lambs naturally infected with *A. phagocytophilum***. a) Intracellular *Anaplasma *organisms (arrows) are present in the lumen of a small vessel. Vasculitis characterised by thickened wall and infiltration of numerous leukocytes is present in this vessel. A few bacteria are observed in the vessel wall, whereas more *Anaplasma *organisms are found in leukocytes in dermis. [Msp2 (P44) immunostain, original magnification, ×1000]. b) Numerous *Anaplasma *organisms (arrows) are found in neutrophils and macrophages infiltrating the dermis. [Msp2 (P44) immunostain, original magnification, ×1000]

Variable numbers of IHC positive organisms were observed in tick bite biopsies from 17/38 lambs (44.7%) and appeared as intracytoplasmic aggregates, known as morulae. The observed organisms were associated with leucocytes in the inflammatory infiltrate in the biopsies and were most often present in neutrophils or macrophages (Fig. 2b). In addition IHC positive organisms were occasionally observed in an extracellular location, either in the lumina of blood vessels or in the adventitial tunic. Bacteria were occasionally located in cells infiltrating the vascular walls of venules or arterioles, usually in Tunica media or Tunica adventitia and rarely in Tunica intima (Fig. 2a). *A. phagocytophilum *organisms were also present in intravascular inflammatory cells in lambs showing vasculitis (Fig. 2a). IHC positive organisms were sometimes observed close to the vessel lumina (Fig. 2b). There was a large variation in the number of IHC positive *A. phagocytophilum *organisms, observed in biopsies between animals, and in different biopsies from the same animal (data not shown). Some biopsies had scattered IHC positive labeling whereas intensively stained aggregates were observed in other lambs. Intensively stained aggregates were mostly observed among inflammatory cell infiltrates of the dermis and subcutis. The control biopsies were IHC negative for *A. phagocytophilum*.

All lambs with IHC positive skin biopsies were also positive for *msp2 *(*p44*) by PCR on blood samples and were seropositive for *A. phagocytophilum *infection. The serological examination gave the best measures of time post infection. Three lambs were seronegative (titre < 40) and had no visible organisms in the skin biopsies examined by immunohistochemistry. Table 1 shows that 20 (80%) of 25 IHC positive skin biopsies were collected from animals with a serum titer ranging from 40 to 1280. Only 5 (20%) of the IHC positive biopsies were collected from the group having titers ranging from 2560 to 5120 (Table 1).

**Table 1:** PCR, Serology and immunohistochemistry on blood, serum and tick bite biopsies, collected from lambs, naturally infected with *A. phagocytophilum*

	Serum titre	≤ 40	40 - 1280	2560 - 5120	Sum
PCR positive blood sample	IHC positive	0	20	5	25
	
	IHC negative	1	21	17	39

PCR negative blood sample	IHC positive	0	0	0	0
	
	IHC negative	2	6	0	8

	**Sum**	3	47	22	**72**

## Discussion

Local infection with *A. phagocytophilum *in tick attachment sites of lambs were characterized by hyperplastic skin changes and inflammatory infiltrates, similar to what is described for tick bite reactions even in the absence of *A. phagocytophilum *infection. Immunohistochemistry showed the presence of *A. phagocytophilum *in approximately 45% of the lambs. These lambs were also positive for *A. phagocytophilum *by PCR on blood and skin biopsies. *A. phagocytophilum *organisms were mainly found in inflammatory cell infiltrates, particularly in PMNs and macrophages of the dermis and subcutis. In the present study, microorganisms were rarely observed in leucocytes in the blood stream of the skin biopsies, whereas Lepidi and coworkers reported that approximately 90% of the infected neutrophils in deep tissues from sheep, humans and horses were seen within vessel lumens [9]. IHC positive organisms were sometimes observed in the mid- and peripheral part of the vessel walls, but rarely in the intimal layer in the present study. The endothelium has earlier been suggested to function as a transition site for transfer of *A. phagocytophilum *to neutrophils that are loosely bound and then released into the blood stream [15]. The present study however indicates that endothelium infection is a rare finding and it does not support the role of endothelium in the pathogenesis of *A. phagocytophilum *infection in lambs, at least at the earliest phases of tick bite inoculation. This stands in contrast to studies reported in mice, for which no morphological images are available [10]. The current study is however, limited in that it is not an experimental study. The field conditions did neither allow control with attached ticks and infectious organisms, nor a longitudinal study of skin lesions, which is best estimated, based upon serum antibody titer, and at this point, endothelium could have played a role.

The very low number (7.9 %) of sero-negative animals (titre < 40), all which were IHC-negative, indicated that most lambs were sampled after seroconversion. Most of the IHC positive skin biopsies were collected from animals with serum titres between 40 and 1280, which may indicate that these had acute infections. However, presence of maternal immunity cannot be neglected, since the half life of maternal antibodies has been estimated to be 17.5 days [27]. Animals with titres between 2560 to 5120, were likely to have seroconverted. The IHC-positive organisms, observed in biopsies from this latter group, may therefore have been associated with an acute infection.

PCR detection of *msp2 *(*p44*) in blood samples showed that at least 86.8 % of the lambs, had *A. phagocytophilum *bacteraemia at the time of sampling. Five *rrs *(16S rRNA gene) variants were encountered. Organisms in biopsies with sequences obtained from variants U02521, M73220, AF336220 and AJ242784 were detected by IHC. Differences in local inflammatory responses to these variants have never been described, but previous studies have shown that different *rrs *(16S rRNA gene) variants of *A. phagocytophilum *can result in different immunological responses and clinical reactions [28]. However, in the present study, no direct relationship between gene variants, serum titre or inflammatory changes were observed. Similar histopathologic findings and inflammatory infiltrates with monocytes and neutrophils were associated with all *rrs *(16S rRNA gene) variants.

Nineteen of 38 sequences (50.0 %) obtained from tick bite sites corresponded to the sequences obtained from the respective ticks. All variants, except GU459257 (isolated from the skin and blood) have previously been isolated from the blood of infected sheep. The sequences obtained from control biopsies and the blood samples however were identical in all lambs, where both sequences were obtained, indicating a haematogenous spread of organisms to the skin. In addition, two PCR positive control biopsies were collected from animals having PCR negative blood, indicating that the organisms may have originated from nearby tick bites or from another infected area due to local dissemination. However, no tick bites were observed within a 20 cm distance from the control biopsies. The reason for these PCR positive control biopsies should be further elucidated as this could comprise a nidus among animals, capable of sustaining persistent infection. Future studies should investigate the extent of cell-to-cell infection in the skin, and how far the infected cells may migrate locally away from tick bites.

Long-term survival in the skin could function as a reservoir during persistent infection and could be a source of transmission to other feeding ticks even in the absence of sustained bacteremia. A previous study reported that the presence of *A. phagocytophilum *in the peripheral blood of small mammals may be short lived and that tissue samples from spleen and ear seemed to be more often infected than blood [29]. The majority of lambs (60.5%) had hyperplastic dermatitis with perivascular to diffuse infiltration of leukocytes. The results also show that the IHC-positive organisms were associated with the leukocytes of the infiltrate at the tick bite site, indicating that PMNs and other inflammatory cells are attracted to the area and may provide a possibility for survival of *A. phagocytophilum *beneath the skin surface. However, the occurrence of persistent skin infections and its role in the transmission of *A. phagocytophilum *to ticks has to be further investigated.

A positive relationship between the degree of inflammation observed by histology and the number of *A. phagocytophilum *organisms detected by IHC, in the same tissue sections was present. However, quantitative studies should be performed to further elucidate this, as control biopsies, also PCR positive for *msp2 (p44)*, did not show inflammatory changes by histology. Neutrophil infiltration can be triggered by tick salivary components and chemotaxins produced by infected neutrophils and other cell types [30,31]. In addition, the presence of pyogenic bacteria such as *Staphylococcus aureus *is commonly associated with tick bites [32], and will also favor the attraction of neutrophil granulocytes.

The present study shows that the majority of IHC positive organisms were present in the inflammatory cell aggregates. In one animal, *rrs *(16S rRNA) genotyping of organisms revealed identical gene variants in the blood and an IHC-positive biopsy from the same animal that differed from the gene variant detected in the attached tick. This indicates that the tick was not the source of infection, or that the infecting variant selectively survived in the lamb. In sections where IHC-positive organisms were detected within the vessel walls, they were usually observed intracellular in leukocytes. A previous study reported that more than 95% of infected cells in tissues were mature neutrophils, based on IHC analysis and that association of *A. phagocytophilum *with vessel walls was rarely observed [9]. However, the direction of migration of organisms needs further investigation. Lesions such as vasculitis and thromboses are reported to be rare findings of *A. phagocytophilum *infection [9], but were present in the tick bite wounds of some lambs in the present study. This may however be caused by tick salivary components or other pathogens like *S. aureus *or streptococci that are likely to be associated with tick bites [32].

In conclusion, whether the endothelium plays a role in the pathogenesis of and establishment of *A. phagocytophilum *infection at the site of tick bite could not be documented by the present study. Other factors or cell types, such as dendritic cells, might be involved, but this was not examined. Controlled experimental studies with serial sampling of infected skin are suggested in order to further elucidate the pathogenesis of this infection during tick attachment.

## Competing interests

The authors declare that they have no competing interests.

## Authors' contributions

EGG performed the sampling, real time PCR, immunohistology, *rrs *sequence analysis and writing of the manuscript. MA participated in the design of the study, performed the histological examination, immunohistological interpretation and created the figures. MA also revised the draft manuscript. KB performed the IFAT. WOT performed the sequencing of *rrs *(16S rRNA gene). JSD participated in the design of the study, provided reagents for IHC and helped in revising the draft manuscript. SS designed the study and supervised the writing of the draft manuscript. All authors read and approved the final manuscript.
